# Grainyhead-like 2 (GRHL2) knockout abolishes oral cancer development through reciprocal regulation of the MAP kinase and TGF-β signaling pathways

**DOI:** 10.1038/s41389-018-0047-5

**Published:** 2018-05-08

**Authors:** Wei Chen, Kyung L. Kang, Abdullah Alshaikh, Saaket Varma, Yi-Ling Lin, Ki-Hyuk Shin, Reuben Kim, Cun-Yu Wang, No-Hee Park, Katharina Walentin, Kai M. Schmidt-Ott, Mo K. Kang

**Affiliations:** 1 The Shapiro Family Laboratory of Viral Oncology and Aging Research, UCLA School of Dentistry, Los Angeles, CA USA; 2 Department of Periodontology, School of Dentistry, Kyung Hee University, Seoul, Korea; 3 Division of Diagnostic and Surgical Sciences, UCLA School of Dentistry, Los Angeles, CA USA; 4 Division of Oral Biology and Medicine, UCLA School of Dentistry, Los Angeles, USA; 5 UCLA Jonsson Comprehensive Cancer Center, Los Angeles, CA USA; 6 David Geffen School of Medicine at UCLA, Los Angeles, CA 90095 USA; 7 Max Delbruck Center for Molecular Medicine, Department of Nephrology, Charité Medical University, Berlin, Germany

## Abstract

Grainyhead-Like 2 (GRHL2) is an epithelial-specific transcription factor that regulates epithelial morphogenesis and differentiation. Prior studies suggested inverse regulation between GRHL2 and TGF-β in epithelial plasticity and potential carcinogenesis. Here, we report the role of GRHL2 in oral carcinogenesis in vivo using a novel *Grhl2* knockout (KO) mouse model and the underlying mechanism involving its functional interaction with TGF-β signaling. We developed epithelial-specific *Grhl2* conditional KO mice by crossing *Grhl2* floxed mice with those expressing CreER driven by the K14 promoter. After induction of *Grhl2* KO, we confirmed the loss of GRHL2 and its target proteins, while *Grhl2* KO strongly induced TGF-β signaling molecules. When exposed to 4-nitroquinoline 1-oxide (4-NQO), a strong chemical carcinogen, *Grhl2* wild-type (WT) mice developed rampant oral tongue tumors, while *Grhl2* KO mice completely abolished tumor development. In cultured oral squamous cell carcinoma (OSCC) cell lines, TGF-β signaling was notably induced by GRHL2 knockdown while being suppressed by GRHL2 overexpression. GRHL2 knockdown or KO in vitro and in vivo, respectively, led to loss of active p-Erk1/2 and p-JNK MAP kinase levels; moreover, ectopic overexpression of GRHL2 strongly induced the MAP kinase activation. Furthermore, the suppressive effect of GRHL2 on TGF-β signaling was diminished in cells exposed to Erk and JNK inhibitors. These data indicate that GRHL2 activates the Erk and JNK MAP kinases, which in turn suppresses the TGF -β signaling. This novel signaling represents an alternative pathway by which GRHL2 regulates carcinogenesis, and is distinct from the direct transcriptional regulation by GRHL2 binding at its target gene promoters, e.g., E-cadherin, hTERT, p63, and miR-200 family genes. Taken together, the current study provides the first genetic evidence to support the role of GRHL2 in carcinogenesis and the underlying novel mechanism that involves the functional interaction between GRHL2 and TGF-β signaling through the MAPK pathways.

## Introduction

Grainyhead-like 2 (GRHL2) is one of the three known mammalian homologs of *Drosophila* Grainyhead (GRH), along with GRHL1 and GRHL3, which are involved in epithelial regeneration and function^1–3^. In addition, we have demonstrated that GRHL2 plays a unique role in control of cellular proliferation and differentiation through transcriptional regulation of its target genes, e.g., *hTERT*, *p63*, and epidermal differentiation complex (*EDC*) genes^4–6^. GRHL2 regulates keratinocyte proliferation; its level declines during replicative senescence or stress-induced senescence, while ectopic GRHL2 expression increases cellular lifespan^4^. Hence, loss of GRHL2 during senescence may lead to reduced hTERT/telomerase activity, resulting in accelerated telomeric attrition and aging. In addition, GRHL2 is a critical determinant of the epithelial phenotype through transcriptional regulation of the relevant effector molecules, e.g., miR-200 family genes and ZEB1, which also determine cellular plasticity^7–9^. GRHL2 has been shown to suppress epithelial–mesenchyme transition (EMT) induced by TGF-β in human mammary epithelial cells^9^, while the mechanisms underlying the functional interaction between GRHL2 and TGF-β are not known.

TGF-β is a growth factor that binds to the TGF-β receptors (types I/II) through auto/paracrine mechanisms and activates transcription cofactors, e.g., Smad2/3 by phosphorylation and nuclear translocation of p-Smad2/3 and Smad4 complex^10^. TGF-β is generally considered a potent growth inhibitor of epithelial cells through induction of diverse cell cycle inhibitory proteins. Hence, it is considered a strong tumor suppressor during the early stage of oral carcinogenesis^11–13^. TGF-β also exhibits malignant effects in established cancers through the induction of EMT and metastasis^14,15^. Thus, TGF-β demonstrates dual roles in the cancer phenotype, which depends in part on the stage of the disease progression.

The current study investigated the functional interaction between GRHL2 and TGF-β signaling and the underlying molecular mechanism. Using epithelial-specific *Grhl2* conditional knockout (cKO) mice, we demonstrate the inhibitory effects of GRHL2 on TGF-β signaling in the epidermis and oral mucosa. *Grhl2* KO did not cause gross phenotypic defects in the epithelia, although there was reduced cell proliferation at the basal cell layers. However, *Grhl2* KO completely abolished the tongue tumor formation after chronic exposure to the chemical carcinogen 4-nitroquinoline 1-oxide (4-NQO), which led to rampant tongue cancer development in the wild-type (WT) mice. Mechanistic investigation revealed, for the first time, that GRHL2 is necessary and sufficient for activation of the Erk1/2 and JNK MAP kinases, which then suppress TGF-β signaling. Taken together, the current study demonstrates evidence for the functional interaction between GRHL2 and TGF-β signaling through MAP kinase pathways, and provides the first genetic evidence to support the role of GRHL2 in the early onset of oral carcinogenesis using the *Grhl2* cKO model.

## Results

### *Grhl2* KO abolishes chemical carcinogen-induced oral carcinogenesis

**Fig. 1:**
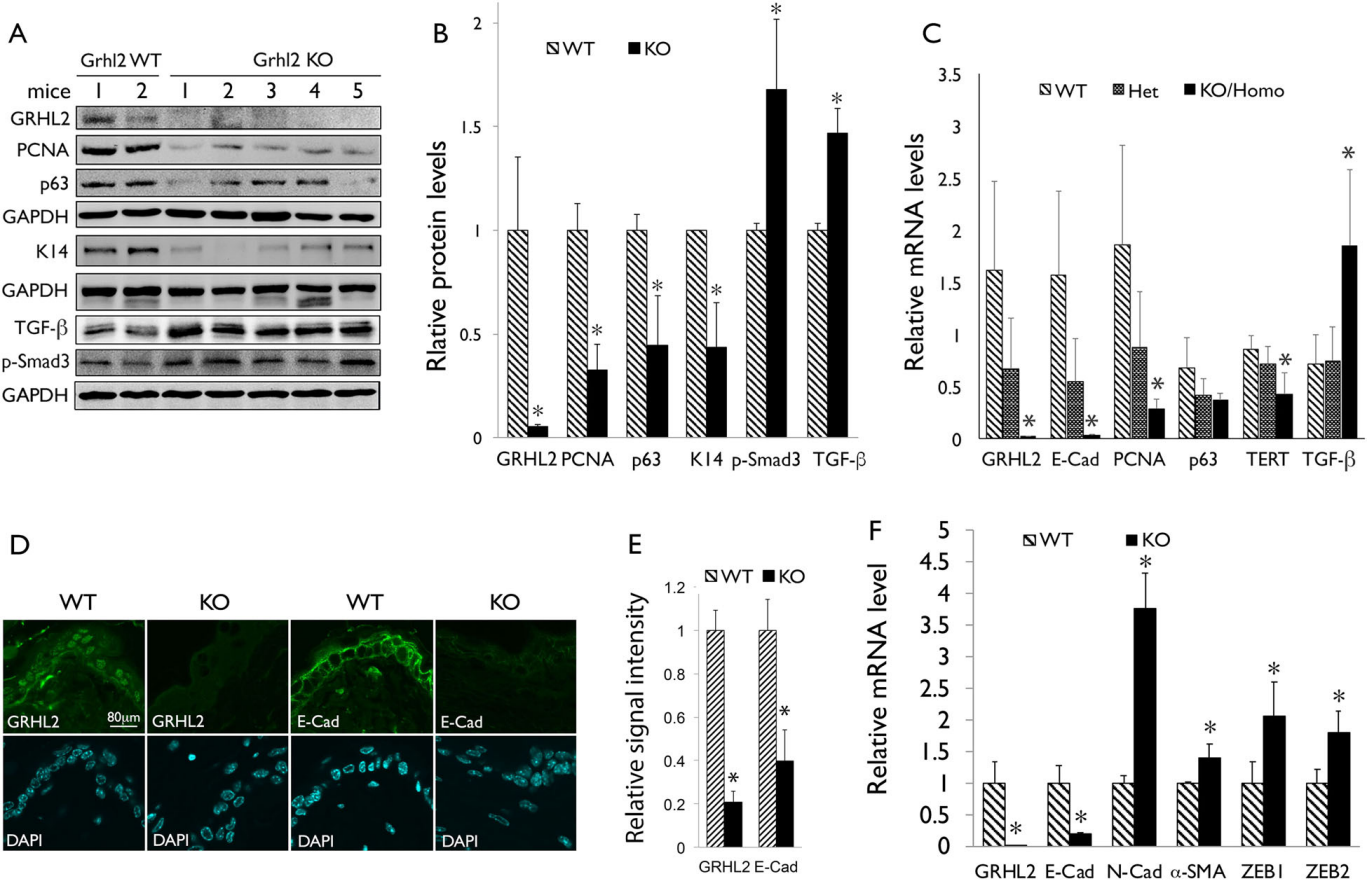
*Grhl2* KO suppresses epithelial phenotype marker genes and induces TGF-β signaling in epithelial tissues in vivo. **a** Western blotting was performed with epidermal tissues isolated from *Grhl2* WT and *Grhl2* KO mice for GRHL2 and various target proteins, e.g., PCNA, p63, K14, and Sox2, as well as TGF-β and p-Smad3. **b** Western blotting signals were quantitated by densitometric analysis and plotted with the mean values for *Grhl2* WT and KO mice groups. Bar indicates mean/SD, *P < 0.05. **c** Quantitative reverse transcription-PCR (qRT-PCR) was performed with total RNAs isolated from *Grhl2* WT, *Grhl2* heterozygote (±) and *Grhl2* KO mouse epidermis. Data were derived from three independent experiments and qRT-PCR assays were performed in triplicates. **d** IFS was performed for GRHL2 and E-Cad in skin epithelia of *Grhl2* WT and KO mice. **e** IFS signals were quantitated and plotted with the mean values for *Grhl2* WT and KO mice groups. **f** qRT-PCR was performed with tongue epithelium harvested from Grhl2 WT mice (*n* = 3) and Grhl2 KO mice (*n* = 3) for various markers of mesenchyme, e.g., N-Cad, α-SMA, ZEB1, and ZEB2. Bar indicates mean/SD. *P < 0.05, statistical significance, compared with WT mice

In order to determine the role of GRHL2 in epithelial tissue regeneration and oral carcinogenesis in vivo, we generated *Grhl2* cKO mice by crossing *Grhl2* floxed (fl/fl) and K14-CreERT, denoted K14/*Grhl2* cKO, which allows for conditional deletion of *Grhl2* by exposure to tamoxifen (Tmx) in an epithelial tissue-specific manner. The resulting mice (*Grhl2* KO) showed absence of GRHL2 expression and downregulation of the GRHL2 target genes, e.g., E-cadherin (E-Cad), PCNA, p63, and TERT (Fig. 1a-c), in the excised skin samples. Upon induction of the Cre expression, loss of GRHL2 and E-Cad expression was detected by immunofluorescence staining (IFS) in the skin epithelia of the *Grhl2* KO mice (Fig. 1d, e). On the other hand, the levels of TGF-β1 and p-Smad3 were notably increased by *Grhl2* KO, suggesting the reciprocal link between GRHL2 and TGF-β1 signaling, although it remains unknown whether TGF-β and p-Smad levels were contributed by epidermis or dermis layers, or both. With *Grhl2* KO, there was also strong induction of mesenchymal markers, e.g., N-cadherin (N-Cad), α-SMA, ZEB1, and ZEB2, in the tongue tissue compared with those of the WT mice, consistent with induction of TGF-β signaling (Fig. 1f).

**Fig. 2:**
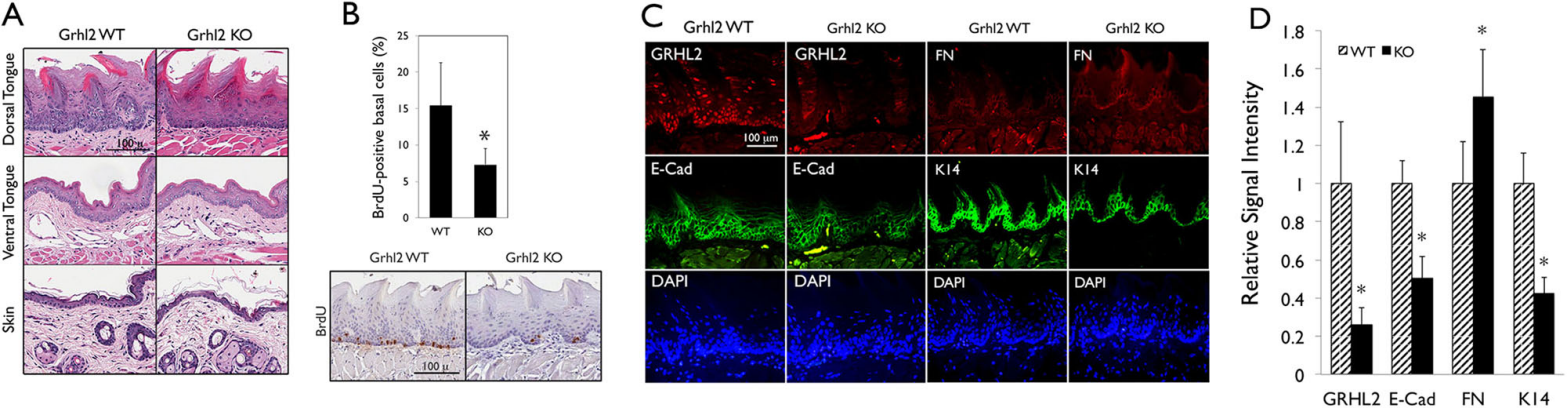
Epithelial-specific *Grhl2* KO leads to retarded basal cell proliferation without gross histological defects in the epithelium. **a** Histology was shown after hematoxylin and eosin (H&E) staining of tongue epithelia (dorsal and ventral surfaces) and skin of *Grhl2* WT and KO mice. **b** *Grhl2* WT and KO mice were labeled with BrdU and the nuclear incorporation was determined by IHC of dorsal tongue epithelium. BrdU-positive cells were counted in each field and plotted as % of all basal cells. Bar indicates mean/SD. *P < 0.05, statistical significance, compared with WT mice. **c** IFS was performed for GRHL2, E-Cad, FN, and K14 in dorsal tongue epithelia of *Grhl2* WT and KO mice. **d** IFS signals were quantitated and plotted with the mean values for *Grhl2* WT and KO mouse groups. Bar indicates mean/SD. *P < 0.05, statistical significance, compared with WT mice

Histologically, *Grhl2* KO mice exhibited reduced cellularity at the basal layers of dorsal tongue epithelium and skin, while maintaining the intact epithelial layers (Fig. 2a). In addition, BrdU incorporation in the basal cell layer of dorsal tongue epithelium was significantly reduced by *Grhl2* KO compared to the WT mice (Fig. 2b), indicating reduced cell proliferation and tissue regeneration by *Grhl2* KO. IFS confirmed complete disruption of GRHL2 expression and significant reduction of GRHL2 target genes, E-Cad and K14, in tongue of *Grhl2* KO mice (Fig. 2c, d). These findings demonstrate that conditional KO of *Grhl2* in stratified epithelium suppressed cell proliferation at the basal cell layer but does not cause gross phenotypic defects in the normal epithelial tissues.

**Fig. 3:**
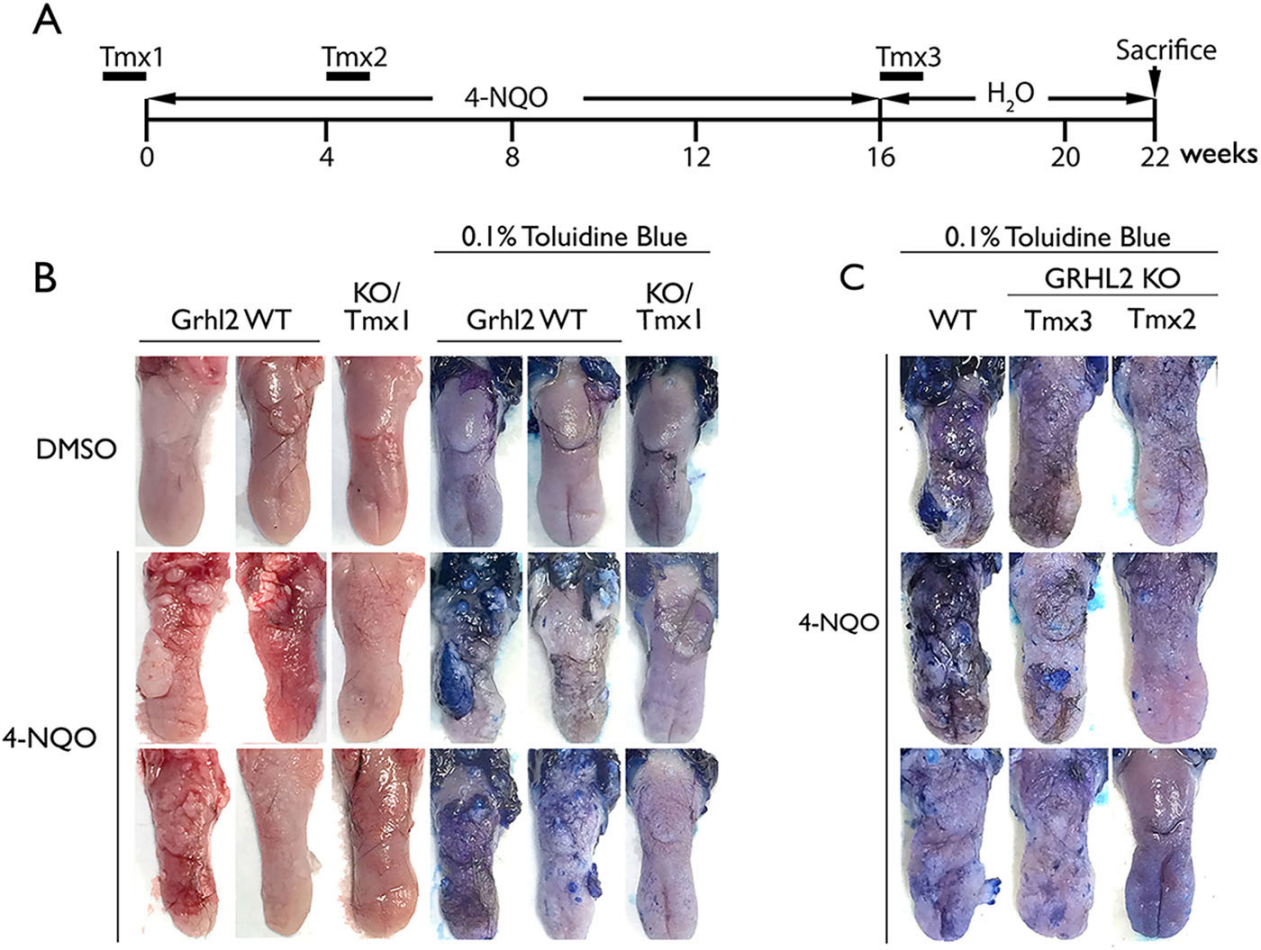
*Grhl2* KO prevents chemical carcinogen-induced tongue carcinogenesis. **a** *Grhl2* KO was induced by tamoxifen (Tmx, 75 mg/kg) administration for 7 consecutive days, immediately prior to 4-NQO (30 μg/ml) exposure (Tmx1, *n* = 6), after 4-week exposure to 4-NQO (Tmx2, *n* = 3), or after 16 weeks of 4-NQO exposure (Tmx3, *n* = 3). These WT mice (*n* = 7) and KO mice (total *n* = 12) were all exposed to 4-NQO for 4 months, and then maintained for additional 6 weeks in drug-free drinking water before they were killed. For comparison, we also included Grhl2 WT (*n* = 5) and KO (*n* = 5) mice exposed to DMSO, which did not elicit tumor development in any mice. **b** *Grhl2* WT and KO (Tmx1) tongues were harvested and stained with 0.1% toluidine blue after exposure to 4-NQO for 16 weeks, and representative specimens are shown. **c** *Grhl2* WT and KO (Tmx2 and Tmx3) tongues were stained with 0.1% toluidine blue after 4-NQO exposure. Representative staining results were shown for tongues of mice in various groups

Our prior study showed the role of GRHL2 in established human oral cancers using in vitro models^7^. In the current study, we used the *Grhl2* KO model to determine the role of GRHL2 in the early stage of oral carcinogenesis in vivo by chronic exposure of mice to 4-NQO. Exposure of oral cavity to drinking water containing varying dose (15–50 μg/ml) of 4-NQO yields precancerous and cancerous lesions on the tongue, oral mucosa, and esophagus with similar molecular pathologic profiles as human oral squamous cell carcinomas (OSCCs)^16,17^. K14/*Grhl2* cKO mice were treated with Tmx (75 mg/kg) at three different time points, as illustrated in Fig. 3a, to induce *Grhl2* KO while the mice were exposed to 16 weeks of 4-NQO. After 16 weeks of 4-NQO treatment, *Grhl2* WT mice exhibited gross tumor nodule formation on the tongue, while *Grhl2* KO mice (Tmx1) displayed complete absence of tumor nodule formation (Fig. 3b, Table 1). In addition, administration of Tmx after 4 and 16 weeks into the 4-NQO exposure (designated as Tmx2 and Tmx3, respectively) also resolved the severity and quantity of oral lesions, revealed by toluidine blue staining (Fig. 3c). Comparison of multiplicity of tumors in each group revealed significant differences between *Grhl2* WT mice vs. the KO mice in Tmx1, Tmx2, and Tmx3 groups (Table 1).

**Table 1:** Tumor nodule formation on tongue epithelium in mice exposed to 4-NQO

Group	Treatment	*n* ^a^	Tamoxifen (week)^b^	Multiplicity of tumor (MOT) per tongue^c^	*P* values^d^
*Grhl2* WT	DMSO	5	None	0	NA
*Grhl2* WT	4-NQO	7	None	3.6	NA
*Grhl2* KO/Tmx1	DMSO	5	−1	0	NA
*Grhl2* KO/Tmx1	4-NQO	6	−1	0	0.0015
*Grhl2* KO/Tmx2	4-NQO	3	4	0.4	0.0139
*Grhl2* KO/Tmx3	4-NQO	3	16	0.7	0.0260

^a^Number of mice per group

^b^Tamoxifen was given for 7 consecutive days to create Grhl2 KO. Number shown represents the timing at which Tamoxifen was given with respect to start of 4-NQO treatment

^c^MOT was calculated by counting the number of visible tumors on the dorsum tongue per mouse

^d^Non-parametric Wilcoxon rank-sum test was performed for each group against those of Grhl2 WT mice treated with 4-NQO

*NA* not applicable

**Fig. 4:**
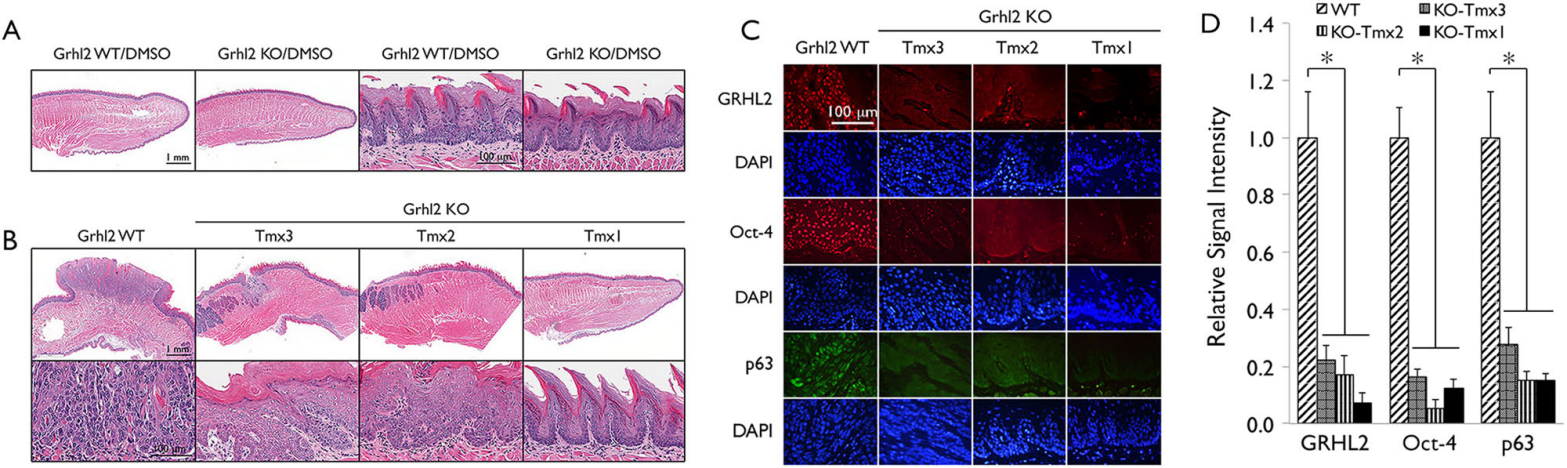
*Grhl2* KO suppresses 4-NQO-induced histopathology in tongue tumors. **a** Gross morphology of tongues from *Grhl2* WT and KO mice exposed to DMSO were shown without altered morphology or histology of the tongue epithelium. **b** In *Grhl2* WT mice treated with 4-NQO, polypoid masses and widespread dysplastic changes are present. A higher-power view shows that the mass is composed of proliferating malignant epithelial islands and has invaded the underlying muscle layer. Keratin formation and a high mitotic index are obvious. Tongue sections from *Grhl2* KO Tmx1 mice show slightly thickened surface epithelium on the tongue. The differentiation is mostly within normal limits. Tongue sections from *Grhl2* KO Tmx2 mice show epithelial hyperplasia with multifocal mild to moderate epithelial dysplasia. Tongue sections from *Grhl2* KO Tmx3 mice show extensive epithelial hyperplasia with widespread dysplastic changes and formation of polypoid masses. Invasion was not noted in any of the *Grhl2* KO mice examined. **c** IHC was performed with tongue tissues from *Grhl2* WT and KO (Tmx1, Tmx2, and Tmx3) mice after 4-NQO exposure for GRHL2, Oct-4, and p63. Nuclei were stained with DAPI. **d** IFS signals were quantitated and plotted with the mean values for *Grhl2* WT and different KO mouse groups after exposure to 4-NQO. Bar indicates mean/SD. *P < 0.05, statistical significance, compared with WT mice

Histologically, 4-NQO treatment of *Grhl2* WT mice induced the formation of polypoid masses with significant cytological atypia, a high mitotic index, and widespread dysplastic changes in the epithelium, while tongues from both *Grhl2* WT and KO mice exposed to dimethylsulfoxide (DMSO) exhibited similar histology with normal squamous epithelium (Fig. 4a). Figure 4b shows a carcinomatous mass on the dorsal tongue of *Grhl2* WT mouse exposed to 4-NQO. The mass is composed of malignant epithelial islands and appears to be invading the muscle layer. In contrast, the carcinogenic effect of 4-NQO was greatly reduced by *Grhl2* KO. When *Grhl2* was knocked out prior to 4-NQO treatment (Tmx1), the carcinogen only resulted in a slightly thicker epithelium (Fig. 4b). The differentiation of the surface epithelium was mostly normal, although focal and very mild cellular alterations were noted on the ventral tongue of some mice. When *Grhl2* was knocked out 4 weeks into 4-NQO treatment (Tmx2), the epithelium developed multifocal mild to moderate epithelial dysplasia with a higher mitotic index. When the gene was knocked out after 16 weeks of 4-NQO treatment (Tmx3), the epithelium developed widespread dysplastic changes and formation of polypoid masses. The masses were generally smaller than those observed in the *Grhl2* WT mice and there was no connective tissue invasion noted in samples evaluated. It suggested that *Grhl2* KO in Tmx3 treatment might still be able to provide some protection again 4-NQO-induced carcinogenesis.

Chronic oral exposure to 4-NQO led to strong nuclear staining for GRHL2 in *Grhl2* WT tumor revealed by immunohistochemistry (IHC), while the tongue epithelium apparently lacked such staining in *Grhl2* KO mice administered with Tmx at all time points (Fig. 4c, d). Likewise, the expression of GRHL2 target proteins, e.g., Oct-4 and p63, were readily detectable in *Grhl2* WT tongue tumor but were significantly suppressed in *Grhl2* KO tissues even exposed to 4-NQO for 4 months (Fig. 4c, d). Western blotting of the tongue tissues confirmed induction of GRHL2 target genes, e.g., TERT and PCNA, after 4-NQO exposure in *Grhl2* WT mice, but these proteins were suppressed in *Grhl2* KO mice. The tongue tissues from *Grhl2* KO mice demonstrated strong elevation of the levels in TGF-β, p-Smad3, and Smad4 compared with those from the WT mice (Supplement Figure S1). These data indicate that GRHL2 expression is required for chemical carcinogen-induced oral cancer development in vivo.

### GRHL2 is required for suppression of TGF-β signaling and MAP kinase activation in OSCC cells

**Fig. 5:**
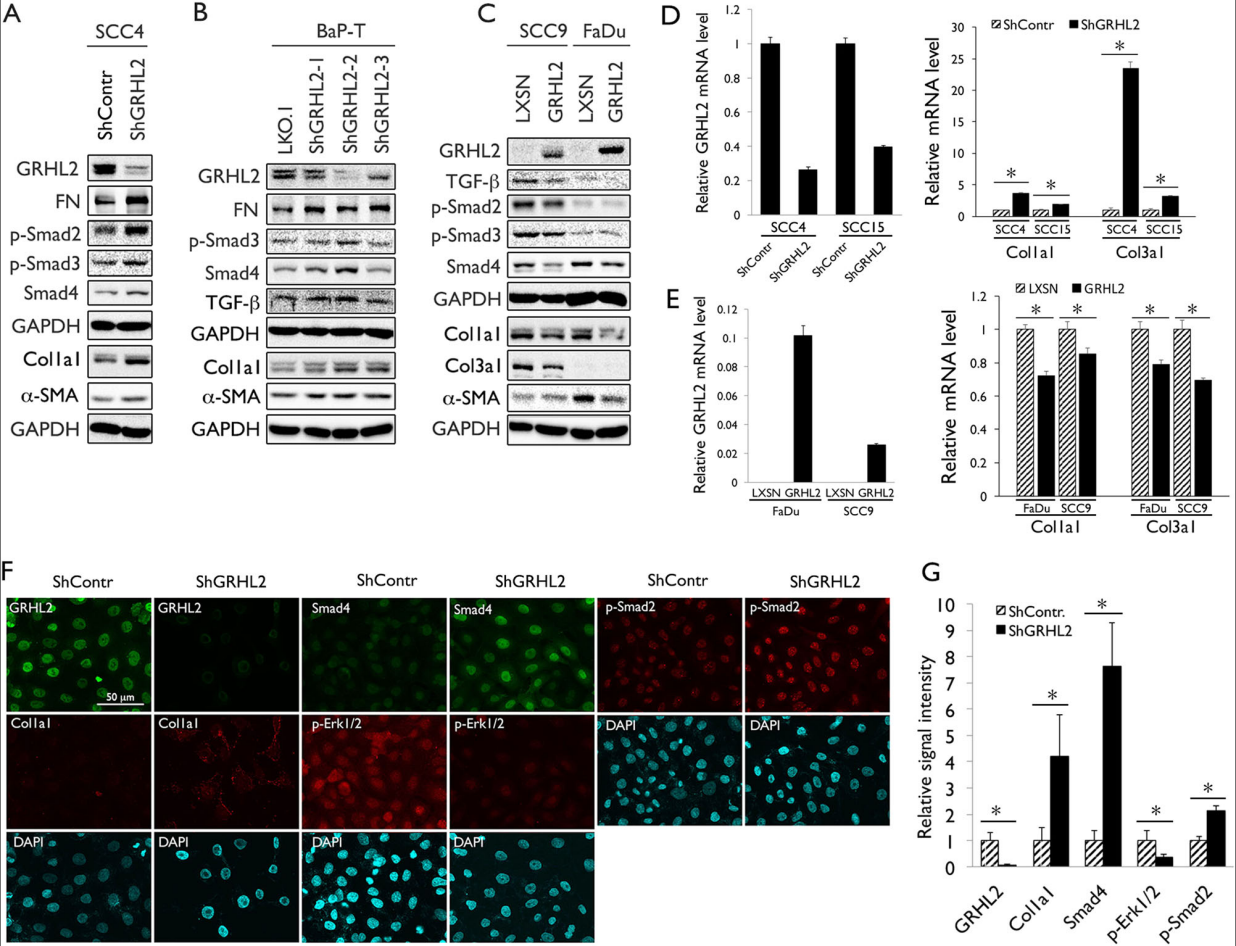
GRHL2 regulates TGF-β signaling in OSCC cells. **a**, **b** Western blotting was performed for TGF-β signaling and the target proteins, e.g., p-Smad2, p-Smad3, Smad4, Col1a1, and α-SMA, in whole-cell extracts of SCC4 and BaP-T cells infected with control lentiviral vectors (ShContr or LKO.1) or those expressing GRHL2 shRNA targeting different regions (ShGRHL2 and ShGRHL2-1,2,3). **c** GRHL2 was overexpressed in SCC9 and FaDu cell lines infected with retroviral vector expressing full-length cDNA of GRHL2 (LXSN-GRHL2) or the empty vector (LXSN) for GRHL2 and TGF-β signaling proteins, e.g., TGF-β, p-Smad2, p-Smad3, Smad4, Col1a1, Col3a1, and α-SMA. **d** qRT-PCR was performed for Col1a1 and Col3a1 in SCC4 and SCC15 cells after GRHL2 knockdown using the lentiviral vector (ShGRHL2). *P < 0.05, significant difference. **e** qRT-PCR was performed in SCC9 and FaDu cells after GRHL2 overexpression for Col1a1 and Col3a1. **f** IFS was performed for GRHL2, Col1a1, p-Smad2, Smad4, and p-Erk1/2 in SCC4 cells stably infected with Lenti-ShGRHL2 or the control vector (ShContr). **g** IFS signals were quantitated and plotted with the mean values for SCC4 cells with *Grhl2* knockdown and its control cells. Bar indicates mean/SD. *P < 0.05, significant difference, compared with SCC4 infected with control vector

A previous study showed the inhibitory role of GRHL2 in TGF-β signaling^8^, which is considered as tumor-suppressive during the early stage of carcinogenesis, in part due to its growth-inhibitory and apoptotic effects^18^. Our in vivo data also demonstrate the reciprocity between GRHL2 and TGF-β signaling, as the levels of TGF-β and p-Smad3 were elevated in *Grhl2* KO mice (Fig. 1). To explore the mechanism by which GRHL2 regulates TGF-β signaling and tumor development, we performed in vitro experiments using established OSCC cell lines, SCC4, SCC15, and BaP-T with stable GRHL2 knockdown through infection with various GRHL2 short hairpin RNA (shRNA) vectors targeting different sequences (pLL3.7-ShGRHL2 for SCC4 and LKO.1-ShGRHL2-1, 2, and 3 for SCC15 and BaP-T; Fig. 5a, b, Supplement Figure S2). These cells were used because they highly express endogenous GRHL2. BaP-T was included herein as a cell line that harbors HPV type 16 DNA^19^. GRHL2 shRNA for SCC4 was fully characterized in our previous study^20^. As expected, GRHL2 knockdown led to increased expression of TGF-β signaling molecules, e.g., p-Smad2/3 and Smad4, as well as TGF-β downstream proteins, e.g., collagen 1a1 (Col1a1) and α-SMA (Fig. 5a, b, d). IFS staining also suggested that loss of GRHL2 elevated TGF-β-signaling molecules in SCC4 cells (Fig. 5f, g). In SCC9 and FaDu cells, which lack endogenous GRHL2 expression, GRHL2 overexpression led to suppression of TGF-β, p-Smad2/3, and Smad4 levels, as well as TGF-β target genes, *Col1a1*, *Col3a1*, and *α-SMA* (Fig. 5c, e, Supplement Figure S3). These results demonstrate the inhibitory effects of GRHL2 in TGF-β signaling in human OSCC cells.

**Fig. 6:**
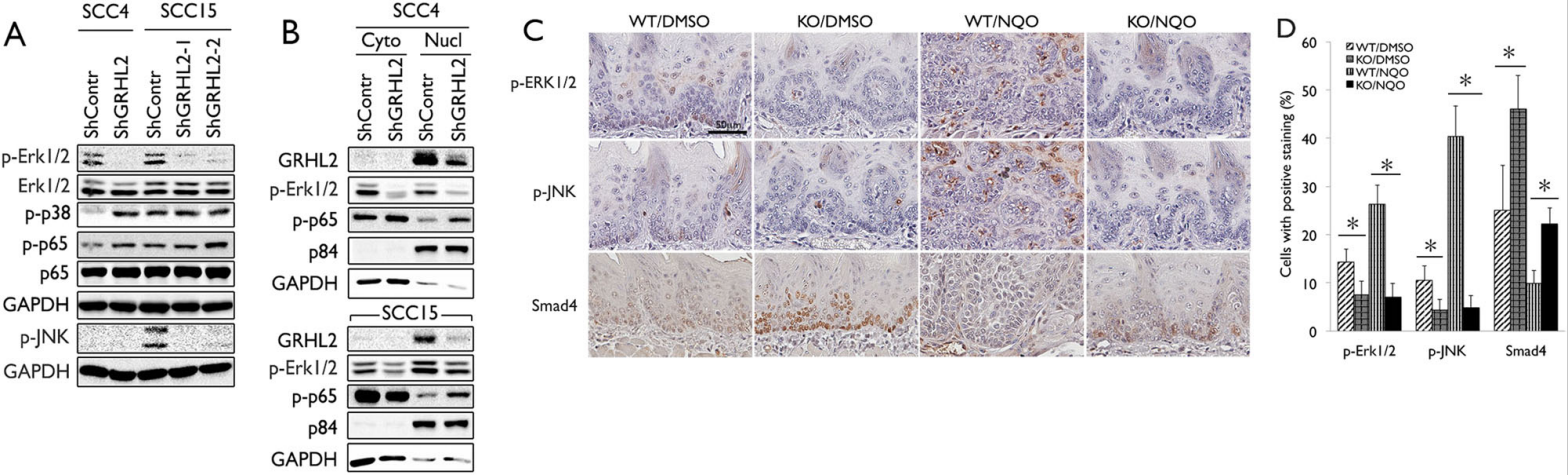
GRHL2 regulates Erk and JNK activation in OSCC cells. **a** In SCC4 and SCC15 cell lines with GRHL2 knockdown, western blotting was performed for p-Erk1/2, p-JNK, p-p38, and p-p65. **b** SCC4 and SCC15 cells with GRHL2 knockdown (ShGRHL2) and the control cells (ShContr) were fractionated into cytoplasmic and nuclear fractions, and western blotting was performed for p-Erk1/2 and p-p65. p84 was used as marker for nuclear fraction, while GAPDH was used as cytoplasmic loading control. **c**, **d** Tongue epithelium from *Grhl2* WT and KO mice exposed to 4-NQO for 16 weeks was stained for p-Erk1/2, p-JNK, and Smad4 by IHC. IHC-positive cells were counted in each field and plotted as % of all cells. Bar indicates mean/SD. *P < 0.05, statistical significance, compared with WT mice

To explore the underlying mechanism by which GRHL2 suppressed the TGF-β pathway, we assessed the role of GRHL2 in activation of the mitogen-activated protein (MAP) kinase signaling, e.g., Erk, JNK, and p38. Prior studies showed functional interaction between MAP kinase and TGF-β signaling pathways, and that Erk proteins blocked nuclear translocation of Smad proteins upon TGF-β activation and induce repressive Smad7^21–23^. With GRHL2 knockdown in SCC4 and SCC15 cells, there was complete absence of p-Erk1/2 and p-JNK, while p38 phosphorylation was increased in SCC4 (Fig. 6A). IFS staining of SCC4/ShGRHL2 cells also revealed diminution of p-Erk1/2 staining in cells (Fig. 5). Thus, GRHL2 may regulate Erk and JNK activation in the MAP kinase-signaling cascade.

With GRHL2 knockdown, the level of phosphorylated p65, indicative of active NF-κB, was notably increased, in SCC4 and SCC15 cells, concomitant with loss of p-Erk (Fig. 6a). In a cell fractionation experiment, GRHL2 knockdown abolished the p-Erk1/2 level in both cytoplasmic and nuclear fractions, and led to elevated p65 phosphorylation in the nuclear fraction (Fig. 6b). Since Erk1/2 is known to suppress NF-κB activation^24,25^, these data further support our finding that GRHL2 plays a role in maintaining the phosphorylation state of Erk1/2 and active MAP kinase signaling. We also surveyed the level of p-Erk1/2 and p-JNK in the tongue tumor tissues in *Grhl2* WT and KO mice exposed to 4-NQO. In both WT and KO mice treated with DMSO, p-Erk1/2 and p-JNK staining was weakly detectable in the tongue epithelium. However, 4-NQO treatment for 16 weeks strongly induced the level of staining in the *Grhl2* WT tissues. The tongue epithelium from *Grhl2* KO mice did not show induction of p-Erk1/2 or p-JNK staining even with 4-NQO treatment (Fig. 6c, d). Smad4 was induced in tongue epithelium from *Grhl2* KO mice compared to WT mice, whereas 4-NQO exposure diminished the Smad4 expression in mice with or without *Grhl2* KO. Western blotting of tongue tissues also revealed strong elevation of the TGF-β level in *Grhl2* KO tissues (Supplement Figure S1). Thus, 4-NQO-induced oral carcinogenesis leads to MAP kinase activation in tongue epithelium, only in the presence of GRHL2 expression, in part through suppressing TGF-β signaling.

### Inhibition of MAP kinase signaling reverses the suppressive effect of GRHL2 on TGF-β signaling

**Fig. 7:**
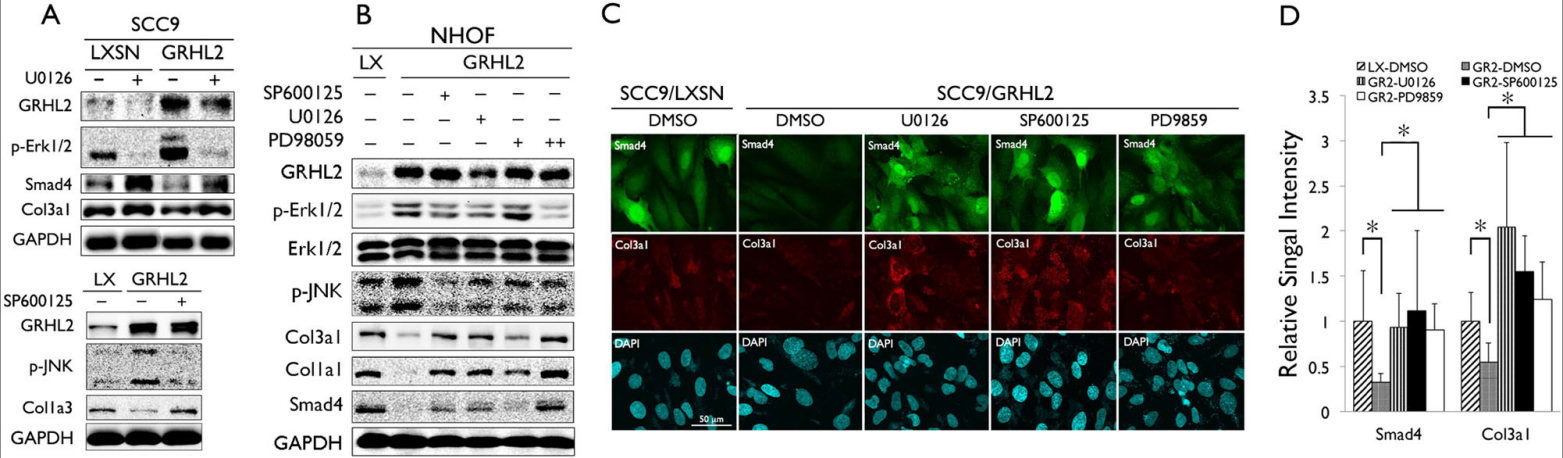
GRHL2 suppresses TGF-β signaling through activation of Erk and JNK MAP kinases. **a** SCC9 cells infected with retroviral vector expressing GRHL2 or the empty control vector (LXSN) were exposed to U0126 (10 μM) for 24 h, and western blotting was performed for GRHL2, p-Erk1/2, Smad4, and C3a1 (upper panels). SCC9/GRHL2 cells were exposed to SP600125 (20 μM) and western blotting was performed for p-JNK and Col1a3 (bottom panels). **b** NHOF infected with retroviral vector expressing GRHL2 or the empty vector (LXSN) were exposed to MAPK inhibitors, SP600125 (20 μM), U0126 (10 μM), or PD98059 (20 and 50 μM), for 24 h and western blotting was performed for GRHL2, p-Erk1/2, p-JNK, and TGF-β target genes (Col3a1, Col1a1, and Smad4). GAPDH was used as loading control for all western blotting analyses. **c** IFS was performed for Smad4 and Col3a1 in SCC9/LXSN or SCC9/GRHL2 cells exposed to DMSO (as controls), U0126 (10 μM), SP600125 (20 μM), or PD98059 (50 μM) for 24 h. **d** IFS signals were quantitated and plotted with the mean values. Bar indicates mean/SD. *P < 0.05, statistical significance, compared with SCC9/GRHL2 cells exposed to DMSO

To test the functional role of GRHL2 in the crosstalk between the TGF-β signaling and MAP kinase signaling pathways, we inhibited p-Erk1/2 in SCC9 cells in which GRHL2 was overexpressed. With exogenous GRHL2, there was clear induction of p-Erk1/2 in SCC9, confirming the positive effects of GRHL2 on the MAP kinase pathway, and suppression of TGF-β signaling molecules, Smad4 and Col3a1 (Fig. 7a). Upon exposure to MEK1/2 inhibitor (U0126), p-Erk1/2 was abolished in cells, and Smad4 and Col3a1 levels were induced, even in the presence of GRHL2 overexpression (SCC9/GRHL2). IFS staining of cells further confirmed re-expression of Smad4 and Col3a1 in SCC9/GRHL2 cells after various MAP kinase inhibitors (U0126, SP600125, and PD9859; Fig. 7c, d).

Ectopic overexpression of GRHL2 in primary NHOF (NHOF/GRHL2) led to increased p-Erk1/2 and p-JNK levels, yet reduced Col3a1, Col1a1 and Smad4 level (Fig. 7b). NHOF was utilized herein because it lacks endogenous GRHL2 expression. We then exposed NHOF/GRHL2 cells to the MAP kinase inhibitors, and assessed the effects on TGF-β signaling, e.g., Smad4, Col3a1, and Col1a1. GRHL2 overexpression in NHOF strongly elevated Erk/JNK phosphorylation and suppressed TGF-β signaling. However, when the Erk/JNK phosphorylation was blocked by the MAP kinase inhibitors, there was re-activation of TGF-β signaling molecules in the presence of GRHL2 overexpression. These novel findings indicate that the inhibitory effect of GRHL2 on TGF-β signaling involves activation of the Erk and JNK signaling of the MAP kinase pathways.

## Discussion

Using the novel epithelial-specific *Grhl2* cKO model, we confirmed the concurrent downregulation of GRHL2 targets and epithelial-specific proteins, e.g., PCNA, p63, β-Cat, K14, and Sox2, as well as upregulation of TGF-β-signaling molecules, e.g., TGF-β1, Smad4, and p-Smad3. Surprisingly, the steady-state histomorphology of cutaneous and oral epithelium remained intact after *Grhl2* KO, although the tissues demonstrated reduced epithelial thickness and cell mitotic activity. Our current finding using the *Grhl2* cKO model is the first genetic evidence to support the role of GRHL2 in primary tumor development from normal oral epithelia. Furthermore, *Grhl2* KO lowered the severity of the tongue lesions compared with *Grhl2* WT mice even after 4 months' exposure to 4-NQO. Hence, the data support the preventive and mitigative effects of *Grhl2* KO on oral dysplastic lesions, and this finding raises the possibility that GRHL2 may be an effective target for oral cancers.

There was strong elevation of TGF-β and its signaling molecules in the tongue tissue of *Grhl2* KO mice when compared with the WT mice. While literature indicates dual roles of TGF-β in cancer progression, TGF-β generally elicits tumor-suppressive effects during the early stages of carcinogenesis by inducing cell cycle regulatory proteins, e.g., p21^WAF1^, p14^ARF^, p16^INK4A^, and p57^KIP^, leading to cellular senescence or apoptosis^26–28^. In addition, loss of Smad4 results in enhanced tumor growth and anti-apoptotic pathways, and increased lymph node metastasis in xenograft models^29^. Hence, the elevated TGF-β signaling in the tongue tissues of the *Grhl2* KO mice would pose strong tumor-suppressive effects, impeding the carcinogenic events induced by 4-NQO. Prior study by Cieply et al. showed the suppressive effects of GRHL2 on TGF-β-induced EMT in mammary epithelial cells^9^. Likewise, in the current study, we demonstrate the suppressive effect of GRHL2 on TGF-β signaling in cultured OSCCs and oral epithelial tissues in mice. In light of primary tumor development, this relationship between GRHL2 and TGF-β signaling further supports the pro-carcinogenic effects of GRHL2 that have been reported by several laboratories, including ours^7,30–35^. This relationship also determines the epithelial plasticity, which plays critical roles in tissue fibrosis, senescence, wound healing, and cancer metastasis. We previously showed that GRHL2 upregulates the expression of miR-200 family genes and other epithelial-specific genes, e.g., p63, K14, E-Cad, and β-catenin, while it suppressed mesenchymal regulators, e.g., fibronectin (FN), N-Cad, ZEB1, and ZEB2^7^. Many of these factors are also woven into the TGF-β-signaling pathways to regulate the epithelial plasticity; thus, the interactions between GRHL2 and TGF-β signaling are antagonistic. Interestingly, a recent study showed inhibitory effects of GRHL2 on p300, a transcription co-activator with histone acetyltransferase activity, during tubulogenesis of kidney epithelial cells^36^. Since p300 functions as a co-activator for TGF-β/Smad signaling^37,38^, p300 suppression may mediate the inhibitory effects of GRHL2 on TGF-β signaling.

The current data also indicate that GRHL2 regulates activation of the Erk and JNK MAP kinase pathways in various cell types, including OSCC cells and NHOF. Previous studies demonstrated the involvement of Erk and JNK in the carcinogenesis process. Immunohistochemical survey revealed an elevated level of Erk in OSCCs with higher grade of histopathology^39^. Ras-raf-MEK-Erk signaling determines cancer cell proliferation and survival, in part through negative regulation of p53 tumor suppressor network^40,41^. A recent study showed JNK activation as the possible etiology of Cetuximab therapy resistance in head and neck squamous cell carcinomas (HNSCCs), which exhibit loss of Smad4 and aggressive phenotype^29^. Hence, activation of MAP kinase signaling has strong pro-carcinogenic effects, in part through its crosstalk with TGF-β signaling. Alternatively, JNK may elicit tumor-suppressive effects, as does GRHL2 in certain pathological context, in part depending on the state of cancer progression^7,42^. A recent study revealed a role of JNK activation for anoikis, a specialized cell death program induced by epithelial cell detachment, which impedes cancer progression^43^. This newly discovered role of JNK may be important for the effects of GRHL2 in sensitizing cells to anoikis during reversion of EMT in human mammary epithelial cells^44^.

Our data indicate that the TGF-β signaling and MAPK pathways reciprocally converge through their functional interactions with GRHL2, which maintains active MAP kinase signaling and suppresses TGF-β signaling. Chemical inhibition of the MAP kinase pathway restored the expression of the TGF-β-signaling molecules in the presence of GRHL2 overexpression. Therefore, the inhibitory effects of GRHL2 on TGF-β signaling appear to be mediated through activation of Erk and JNK signaling. We also found increased phosphorylation and nuclear translocation of p65 in cells with GRHL2 knockdown, which exhibited reduction in the p-Erk level. This provides another layer of evidence to support the role of GRHL2 in Erk regulation as prior studies demonstrate the inhibitory effects of Erk on NF-κB activation^24,45^. Direct effect of GRHL2 in NF-κB signaling is not known, but our data at least suggest inverse correlation between the two factors, possibly through Erk signaling.

**Fig. 8:**
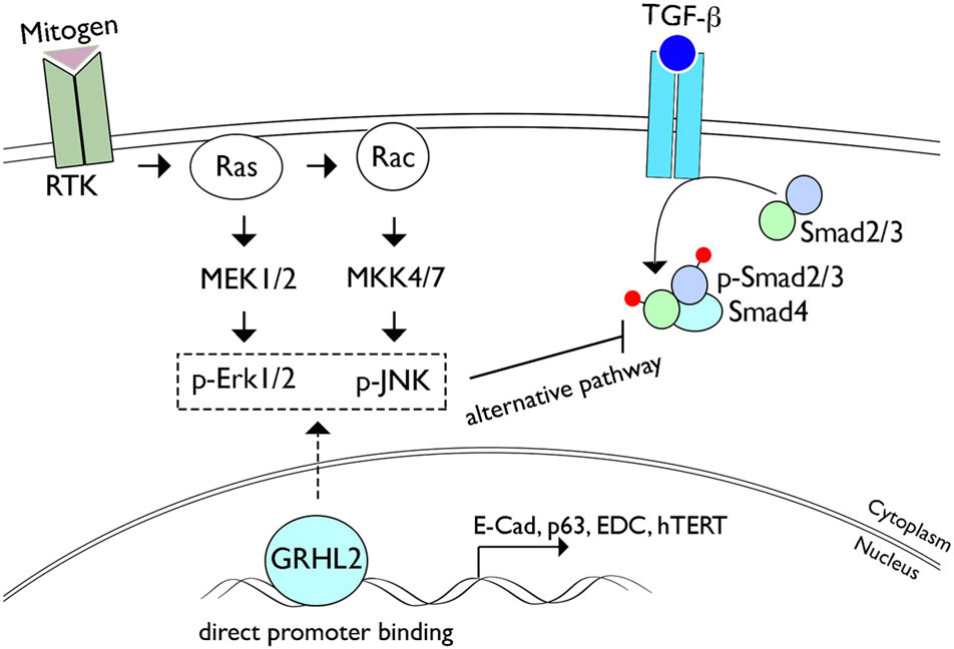
Schematic representation for functional interaction between GRHL2 and TGF-β signaling through MAP kinase pathways. Mechanistic scheme is drawn to depict the functional interaction between GRHL2 and TGF-β signaling through Erk and JNK MAP kinase signaling. The current study identified an alternative pathway by which GRHL2 regulates TGF-β signaling through activation of the MAP kinase signaling pathways. This pathway is distinct from the transcriptional regulation of the target genes, e.g., E-cadherin, hTERT, p63, and miR-200 family genes, by direct GRHL2 binding at the promoter regions

Although TGF-β can directly activate the MAP kinase pathways, e.g., Erk, JNK, and p38, MAP kinase activation via TGF-β-independent mechanism can suppress the TGF-β signaling pathway. For instance, EGF-activated Erk can directly phosphorylate Smad3 at Ser 207, Ser 203, and Thr 178 residues, which suppress Smad transcriptional activity^46^. Furthermore, Erk can directly phosphorylate Smad4 and interfere with Smad4 interaction with R-Smads, causing reduced transcriptional activity of TGF-β target genes^47^. Hence, aberrant GRHL2 overexpression in OSCCs may abrogate the TGF-β-mediated tumor-suppressive effects through these biochemical effects of MAP kinases. We propose a mechanistic model that illustrates the functional interaction between GRHL2 and TGF-β signaling pathway through Erk and JNK signaling (Fig. 8). Generally, GRHL2 regulates epithelial phenotype through direct transcriptional control of its target genes, e.g., E-Cad, p63, hTERT, miR-200 family genes, and EDC genes^4,5,7^. The current study revealed an alternative pathway, in which GRHL2 determines epithelial phenotype through activation of the MAP kinase signaling, which then suppresses Smad-dependent TGF-β signaling molecules. Further research will elucidate the details by which GRHL2 controls the Erk and JNK MAP kinases.

## Materials and methods

### Cells and cell culture

SCC4, SCC9, FaDu, and SCC15 cell lines were purchased from the ATCC (Manassas, VA) and were cultured in DMEM/Ham’s F-12 (Invitrogen, Carlsbad, CA) supplemented with 10% fetal bovine serum (FBS) and 0.4 µg/ml hydrocortisone. The cell lines have been authenticated and tested for mycoplasma contamination. To overexpress GRHL2 in cells, we utilized a retroviral vector (LXSN-GRHL2) expressing GRHL2 according to methods described elsewhere^7^. Endogenous GRHL2 was knocked down in SCC4 using the lentiviral vector (LV-ShGRHL2) expressing shRNA against the GRHL2 target sequence or the control lentiviral vector (LV-ShContr), as described previously^4,20^. GRHL2 was also knocked down in SCC15 and BaP-T cells using commercial GRHL2 shRNA vectors (ShGRHL2-1, -2, -3) targeting different *Grhl2* sequences (Origene, Rockville, MD).

### Generation of *Grhl2* cKO mice

Epithelial tissue-specific *Grhl2* cKO mice (denoted K14/*Grhl2* cKO) were generated by crossing *Grhl2* floxed (fl/fl) mice^48^ with the K14-CreERT mice (Jackson Laboratory, Bar Harbor, ME)^49^. *Grhl2* cKO was achieved with intraperitoneal (i.p.) administration of Tmx (75 mg/kg) in 8-week-old K14/*Grhl2* cKO mice for 7 consecutive days to induce homozygous deletion of *Grhl2* in epithelial tissues.

Eight-week-old WT and KO mice (male and female, 20–26 g) were housed and monitored in individually ventilated case system with ad libitum access to water and food. Animal experiments were performed according to the protocol approved by the UCLA Institutional Animal Care and Use Committee. These WT and KO mice were randomly allocated into different groups exposed to 4-NQO or DMSO. To induce tongue SCC development, *Grhl2* WT (without Tmx) and *Grhl2* KO mice were exposed to 4-NQO (Sigma-Aldrich, St. Louis, MO) dissolved in DMSO and diluted in drinking water to a final concentration of 30 μg/ml. For the 4-NQO assay, we only included *Grhl2* WT and KO, without heterozygotes. *Grhl2* WT or *Grhl2* KO mice were maintained with or without 4-NQO in drinking water for 16 weeks, followed by 6 weeks of normal drinking water, as described previously^50^. We also administered Tmx after 4 or 16 weeks of 4-NQO treatment. These mouse groups were labeled as Tmx2 and Tmx3, respectively, to designate the time at which Tmx was given to induce *Grhl2* KO. Tmx1 denoted the *Grhl2* KO mice in which Tmx was administered right before 4-NQO treatment. In total, there were *Grhl2* WT (*n* = 7), *Grhl2* KO/Tmx1 (*n* = 6), *Grhl2* KO/Tmx2 (*n* = 3), and *Grhl2* KO/Tmx3 (*n* = 3; Table 1). In addition, we included *Grhl2* WT mice (*n* = 5) and *Grhl2* KO mice (*n* = 5), which were exposed to DMSO, as controls. The number of mice were determined by pilot experiments between *Grhl2* WT and *Grhl2* KO.

### Quantitative reverse transcription-PCR analysis

Biopsies from mouse dorsal skin were punched and immediately snap-frozen in liquid nitrogen and stored at −80 °C until further processing. Total RNA was isolated using the TRIzol Reagent (ThermoFisher Scientific, Waltham, MA). DNA-free total RNA (5 μg) was used for reverse transcription (RT) reaction followed by quantitative PCR (qPCR) with LC480 SYBR Green I master using universal cycling conditions in LightCycler® 480 (Roche, South San Francisco, CA). The primer sequences were obtained from the Universal Probe Library (Roche). The PCR cycling conditions were 45 cycles of 10 s at 95 °C, 45 s at 55 °C, and 20 s at 72 °C. Second derivative Cq value determination method was used to compare the fold differences. Cp is the cycle at which the threshold is crossed. Experiments were performed in triplicates.

### Western blotting

Whole-cell extracts (WCEs) from the cultured cells and mouse tissues (skin and tongue) were isolated using the lysis buffer (1% Triton X-100, 20 mM Tris-HCl pH 7.5, 150 mM NaCl, 1 mM EDTA, 1 mM EGTA, 2.5 mM sodium pyrophosphate, 1 μM β-glycerophosphate, 1 mM sodium orthovanadate, 1 mg/ml PMSF). WCEs were then fractionated by SDS-PAGE and transferred to Immobilon membrane (Millipore, Billerica, MA), which were incubated successively with the primary and the secondary antibodies, and exposed to the chemiluminescence reagent (Amersham Pharmacia Biotech, Piscataway, NJ) for signal detection. Each experiment was performed in triplicate.

### IHC and IFS analyses

Paraffin-embedded histological sections were stained with H&E for determination of the histological changes in epidermal and tongue epithelium in *Grhl2* WT and KO mice, and were analyzed without blinding by an oral pathologist. Mouse oral mucosa and skin were fixed in 4% (wt/vol) paraformaldehyde at 4 °C for 24 h. Samples were embedded in paraffin, sectioned at 4 μm thickness, and stained as described previously^7^. Numbers of positive staining cells were counted and plotted as % of all cells in at least 10 fields in each slide.

For IFS, cells were cultured in Nunc™ Lab-Tek™ II Chamber Slide™ System (ThermoFisher Scientific) to reach 70–80% confluence and fixed in 2% paraformaldehyde for 20 min. Cells were permeabilized with 0.2% Triton X-100 in phosphate-buffered saline (PBS) for 10 min, and then blocked for 1 h in PBS containing 2% FBS, and incubated overnight at 4 °C with the primary antibody. After three washes with PBS, cells were incubated with the secondary antibody for 1 h. Slides were mounted in Prolong Gold w/DAPI (Invitrogen). Images were captured on an Olympus epifluorescence inverted microscope (Olympus, Cypress, CA). IFS signals were quantified via Image J software and the corrected total cell fluorescence was calculated.

### BrdU labeling

Keratinocyte proliferation was measured by intraperitoneal injection of BrdU (0.1 mg/g body weight in 0.9% NaCl) 1 h before being killed. BrdU incorporation was detected by IHC of paraffin-embedded sections using anti-BrdU monoclonal antibody (Santa Cruz Biotechnology, Santa Cruz, CA). The number of BrdU-positive and total basal keratinocyte cells was counted in at least 10 areas in each slide.

### Reagents

The following primary antibodies were used in this study: GAPDH, ZEB1, E-Cad, FoxM1, HELLS, Cyclin B1, Cyclin D1, Cyclin A, K14, β-catenin, ZEB1, BrdU, and p63 from Santa Cruz Biotech; GRHL2 (Abnova, Taipei City, Taiwan; H00079977-A01); N-Cad from BD Biosciences (San Jose, CA); FN and Snail from Sigma-Aldrich; p-Smad3 (ser423/425), p-Smad2 (ser465/467), Smad4, p-p38 (Thr180/Tyr182), p-Erk1/2 (Thr202/Tyr204), p-JNK (Thr183/Tyr185), p-p65 (Ser536), and Oct-4 from Cell Signaling Technology Inc. (Danvers, MA); hTERT and Sox2 from Abcam (Cambridge, MA); TGF-β from Novus (Littleton, CO); and PCNA from Calbiochem (San Diego, CA). Secondary peroxidase-conjugated anti-rabbit or anti-mouse antibodies were from Jackson ImmunoResearch Laboratories Inc. (West Grove, PA). Tmx and 4-NQO were purchased from Sigma-Aldrich, while TGF-β1 was from PeproTech Inc. (Rocky Hill, NJ). JNK selective inhibitor SP600125 was purchased from Sigma-Aldrich and MEk1/2 inhibitors U0126 and PD98059 were from Cell Signaling Technology Inc.

### Statistical analysis

Statistical analysis was performed using Student’s *t-*test (two-tailed) for the quantitative reverse transcription-PCR gene expression and BrdU labeling, western blotting, and immunostaining experiments. *P* values < 0.05 were considered significant. All data are expressed as mean ± SD. Statistical differences between the number of tumor nodules in WT mice and different groups of KO mice exposed to 4-NQO were evaluated by the non-parametrical Wilcoxon rank-sum test for each pair of groups. The number of mice in each group was determined by phenotypic changes observed with *Grhl2* deficiency in the cell lines^7,20^.

## Electronic supplementary material

Figure S1

Figure S2

Figure S3
