# Supplementary figures and images for: Grainyhead-like 2 (GRHL2) knockout abolishes oral cancer development through reciprocal regulation of the MAP kinase and TGF-β signaling pathways
Source: Oncogenesis. 2018 May 8;7(5):38. doi: 10.1038/s41389-018-0047-5 (PMC5938237; doi:10.1038/s41389-018-0047-5)

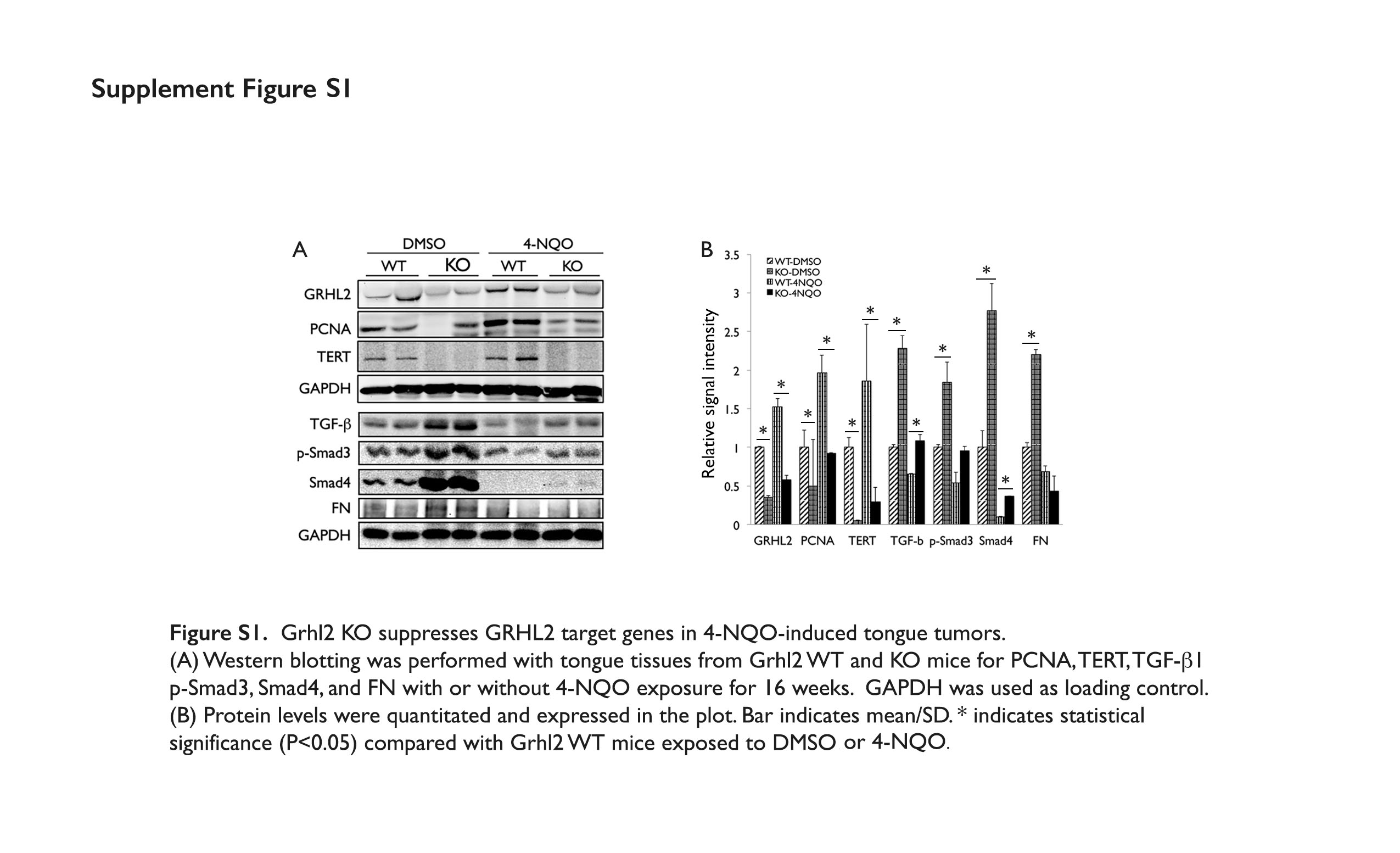

Supplement: Supplementary file 1 — Figure S1 [file 41389_2018_47_MOESM1_ESM.jpg]

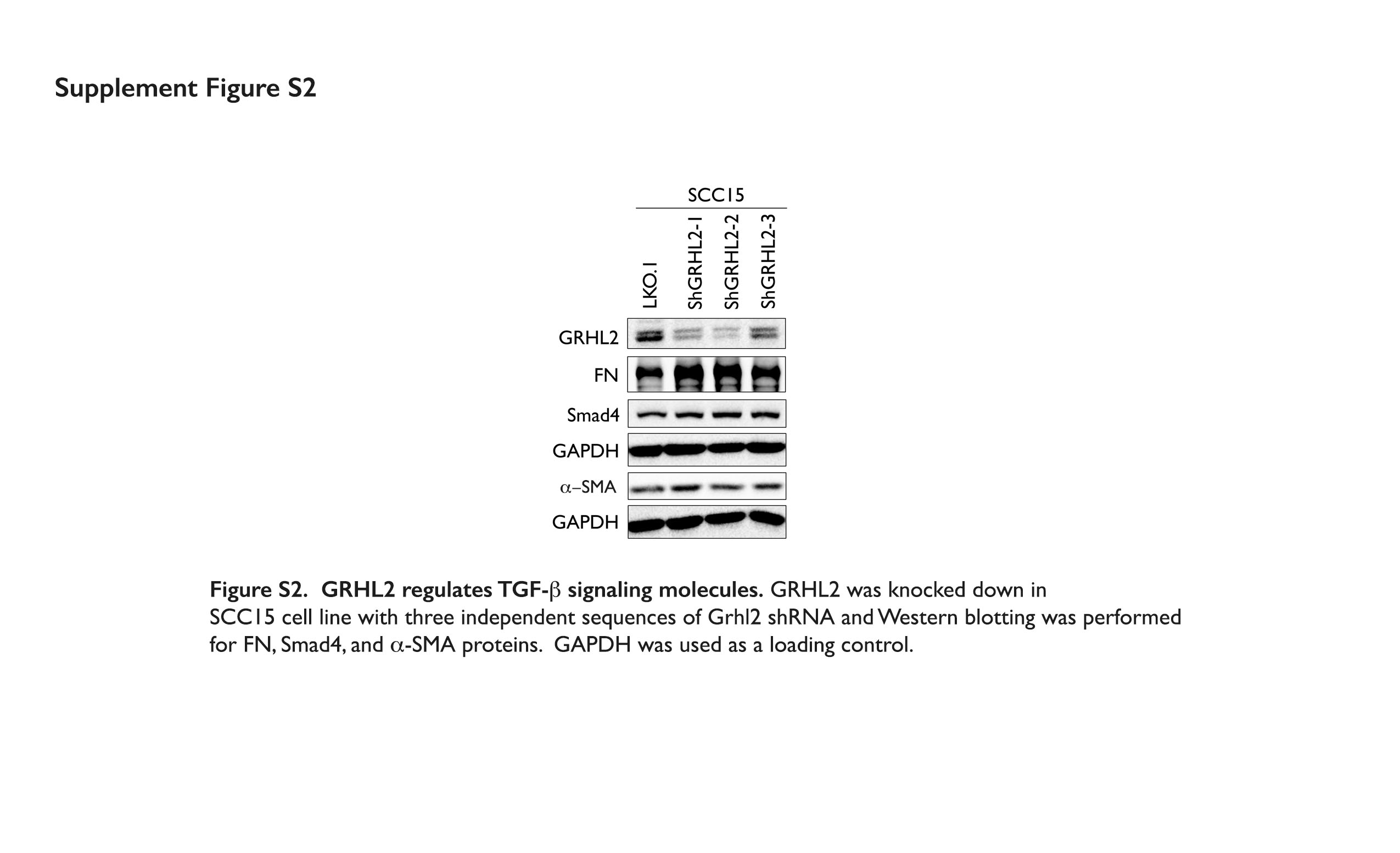

Supplement: Supplementary file 2 — Figure S2 [file 41389_2018_47_MOESM2_ESM.jpg]

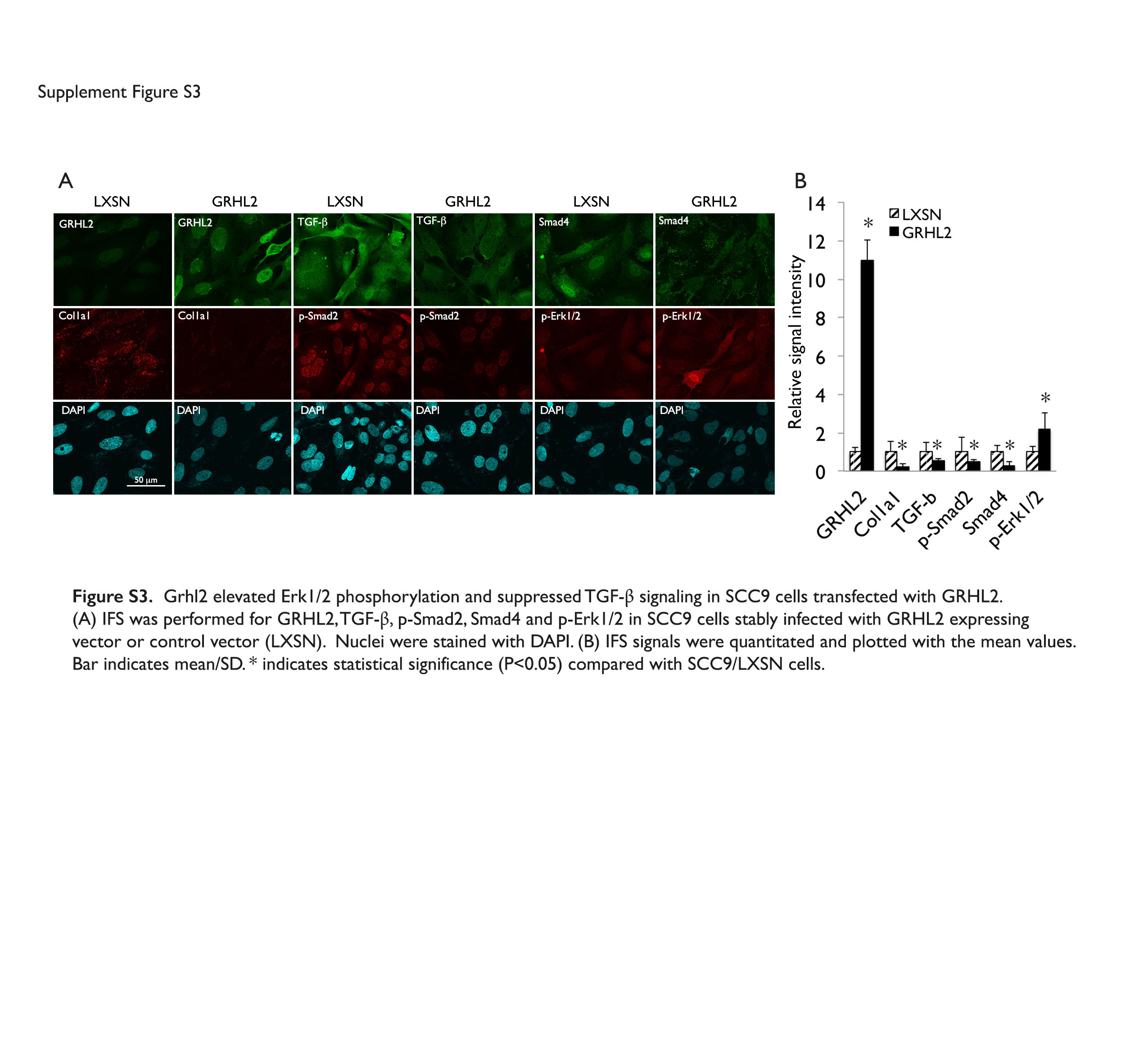

Supplement: Supplementary file 3 — Figure S3 [file 41389_2018_47_MOESM3_ESM.jpg]
